# Determining the Long-Term Skid Resistance of Steel Slag Asphalt Mixture Based on the Mineral Composition of Aggregates

**DOI:** 10.3390/polym15040807

**Published:** 2023-02-06

**Authors:** Kuo Ji, Changchun Shi, Jing Jiang, Yaogang Tian, Xiaowei Zhou, Rui Xiong

**Affiliations:** 1 School of Materials Science and Engineering, Chang’an University, Xi’an 710064, China; 2 Engineering Research Center of Pavement Materials, Ministry of Education of People’s Republic of China, Chang’an University, Xi’an 710064, China

**Keywords:** steel slag, steel slag asphalt mixture, aggregate mineral composition, long-term skid resistance, differential polishing, surface texture richness

## Abstract

This study intends to predict the long-term skid resistance of steel slag asphalt mixture (SSAM) from the mineral composition of the aggregates. The polished stone value (PSV) and mineral composition of the aggregates were assessed using the accelerated polishing test and X-ray diffraction, respectively. The hardness (H) and surface texture richness (STR) of the aggregates were calculated from the mineral composition of the aggregates, and then a multivariate linear model was established between PSV and H and STR. The British pendulum number (BPN) and three-dimensional morphology of the SSAM were then evaluated using a British pendulum and a pavement laser scanner, respectively. Finally, an exponential relationship was established between BPN, aggregate PSV, and various aggregate amounts of SSAM. The results show that steel slag with H, STR, and PSV was better than natural aggregates and can significantly improve the skid resistance of pavement, but the relationship between steel slag content and long-term skid resistance of SSAM was not linear, and SSAM with 50% steel slag content had the best skid resistance. The mathematical model developed can predict the long-term skid resistance of SSAM from the mineral composition of the aggregates. The model can be used by designers to predict the long-term skid resistance of steel slag asphalt pavements at the design stage and thus better determine the proportion of steel slag to other aggregates.

## 1. Introduction

Skid resistance is an important property of asphalt pavement, and a high level of skid resistance is a requisite for road safety. The skid resistance of asphalt pavement is mostly determined by the skid resistance of the road surface aggregates. Consequently, the use of aggregate with a high skid resistance is the key to enhancing the skid resistance of asphalt pavement.

Steel slag is one of the byproducts of the steelmaking process, and China has more than two billion tons of it in reserve [1]. Steel slag possesses superior polishing resistance [2,3] and adhesion qualities [4,5,6] compared to natural aggregates, as well as a lower crushing value [7], Los Angeles abrasion value [8], and needle-like particle content [9]. In addition, the steel slag has a more regular grain shape and richer edges and corners [10,11]. Clearly, steel slag provides significant benefits for enhancing the skid resistance of asphalt pavement [12,13].

Numerous studies have shown the positive effect of steel slag on the skid resistance of asphalt pavements, and this has been confirmed by long-term monitoring of pavements in the field. Liapis [14] monitored a steel slag asphalt pavement constructed in 2008 for 30 months and discovered that its skid resistance was consistently superior to that of natural aggregate asphalt pavement. Kowalski [15] also found that the skid resistance of steel slag asphalt pavement was 10–15% greater than that of conventional aggregate asphalt pavement. Oluwasola [16] examined the usage effects of steel slag and granite in asphalt pavement and found that steel slag asphalt pavement was superior to granite asphalt pavement in terms of both skid resistance and rut resistance. Moreover, according to pertinent publications [17,18], the service performance of stone mastic asphalt with steel slag was superior to that of dense-graded asphalt concrete with steel slag. In addition, steel slag had a positive impact on the service performance of open graded friction courses (OGFC). Steel slag may not only increase the skid resistance of OGFC, but also the drainage capacity of the OGFC surface, hence decreasing the likelihood of cars skidding in wet conditions [19,20].

Due to its complicated contour form and superior mechanical qualities compared to natural aggregates [21], steel slag is also utilized to improve the skid resistance of micro-surfacing. The use of steel slag in micro-surfacing can raise the positive texture of the micro-surfacing and enhance its wear resistance [22]. Keymanesh et al. [23] discovered that when micro-surfacing included 50% steel slag, the wear resistance rose by 19.0%. The research of Zalnezhad [24] revealed that steel slag not only exhibited exceptional resistance to wheel wear, but also exhibited remarkable resistance to water damage on the micro-surfacing.

The steel slag’s long-term skid resistance is strong, but its volume stability is low [25]. Improper treatment of the asphalt mixture may result in expansion cracking [26], and the high porosity of steel slag will increase the asphalt content of asphalt concrete [27]. Consequently, steel slag is commonly combined with other aggregates to reduce the volume expansion and asphalt content of the mix. Presently, the academic consensus is that basalt should be used for road building. However, basalt distribution in China is exceedingly unequal, and China’s annual demand for aggregates surpasses 18 billion tons. China’s building demands are tough to satisfy using basalt. Although the overall performance of limestone is inferior to that of basalt, it is inexpensive and extensively available in China; hence, it is commonly employed for road construction there. In conclusion, steel slag can significantly improve the long-term skid resistance of pavement, and the combination of steel slag and limestone is in high demand for future road construction in China. However, it is not enough for designers to know that the combination of steel slag and limestone can improve the skid resistance of conventional pavements. Being able to accurately predict the long-term skid resistance of steel slag asphalt pavements before they are laid is extremely important for the rational design of steel slag asphalt pavements, but there is a lack of research in this area.

For these reasons, this work employed limestone and steel slag to produce steel slag asphalt mixture (SSAM) and attempted to estimate the long-term skid resistance of SSAM based on the aggregates’ mineral composition. The Polished Stone Value (PSV) test was used to determine the aggregate’s resistance to long-term polishing. X-ray diffraction was then used to evaluate the aggregate mineral composition. The skid resistance of SSAM was then evaluated using a three-wheel wear device and a pavement laser scanner. The mathematical link between the mineral composition of aggregates and the long-term skid resistance of SSAM was determined.

## 2. Materials and Methods

### 2.1. Materials

The materials utilized in this study include aggregates, mineral filler, asphalt binder, and fiber. The aggregates include steel slag, limestone, basalt, and granite. Steel slag is a converter steel slag manufactured by China Binxin Steel Co., Ltd. Limestone, basalt, and granite are from Lingshou County, China. The mineral powder is limestone mineral powder, produced in Lingshou County, China. The asphalt binder is an SBS modified asphalt binder manufactured by China Maoming Fuwei Chemical Co., Ltd. the fiber is lignin fiber from Tangshan, China, and the amount of fiber in the asphalt mixture is 0.4% (mass ratio). The performance of aggregates was evaluated in accordance with the Chinese standard JTG E42-2005. Table 1 displays the physical performance of aggregates.

### 2.2. Specimens Preparation

#### 2.2.1. PSV Specimens

The aggregates of 9.5–13.2 mm were selected, and epoxy resin mortar was used to prepare the PSV specimens for the four aggregates according to the requirements of T 0321 of Chinese standard JTG E42-2005. The freshly prepared specimens were baked for 3 h at 40 °C and then allowed to cool naturally for 9 h before demolding. 

#### 2.2.2. SSAM specimens

The SSAM specimens were produced using the Marshall molding method in accordance with the requirements for hot mix asphalt in Chinese standard JTG F40-2004. The specimen size was 500 mm × 500 mm × 50 mm. The asphalt mixture gradation type is the midpoint of the SMA-5 gradation range specified by the NCHRP of the United States. Two aggregates, limestone and steel slag, were used to prepare the asphalt mixture. The limestone with a particle size range of 2.36–9.5 mm was substituted with an equivalent mass of steel slag in the proportions of 0%, 25%, 50%, 75%, and 100%, respectively.

### 2.3. Methods

Experiment 1: The PSV specimens of the four aggregates were polished on an accelerated polishing machine (JM-3, China) for a total of 7 cycles, with each cycle being polished 40,000 times. After each polishing cycle, the PSV value of the specimen was tested using a British pendulum (BM-III, Chian). Three PSV specimens were made for each aggregate, and the PSV was taken as the average of the three specimen test results.

Experiment 2: The aggregate particles were recovered from PSV specimens of steel slag and limestone after 7 polishing cycles; these were then cleaned and dried together with unpolished aggregate particles. All aggregate particles were gold-blasted, and then the surface morphology was observed using a scanning electron microscope (S-4800, Japan).

Experiment 3: The mineral composition of aggregate was determined by X-ray diffraction (D8 Advance, Karlsruhe, Germany). The experiment was carried out under the conditions of 20 kV voltage, 10 mA current, and 15–70° range on 2θ. The peaks of X-ray diffraction spectra were identified by JADE 6.0 software, and quantitative mineral calculations were carried out.

**Figure 1:**
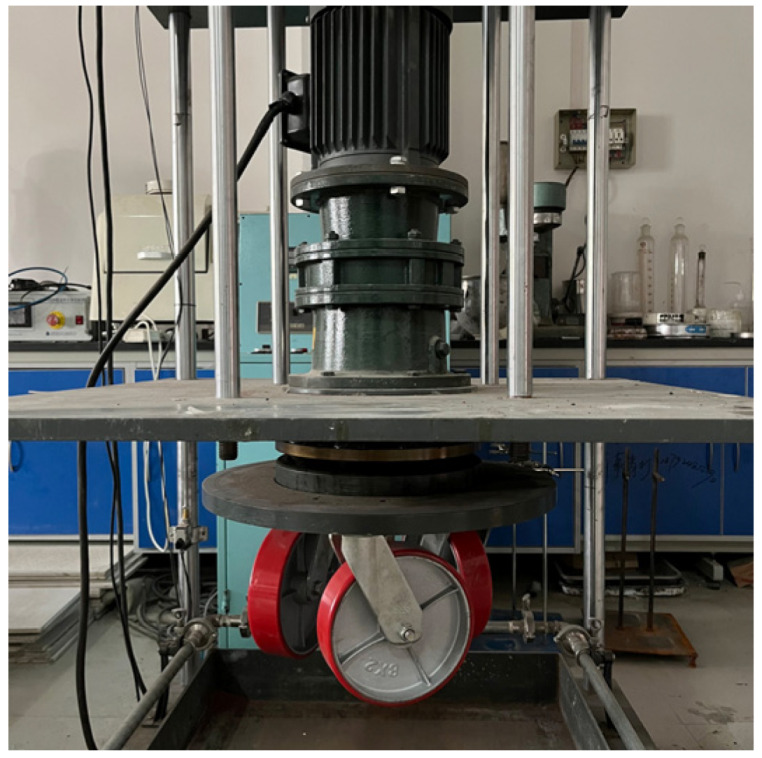
Three-wheel wear device.

**Figure 2:**
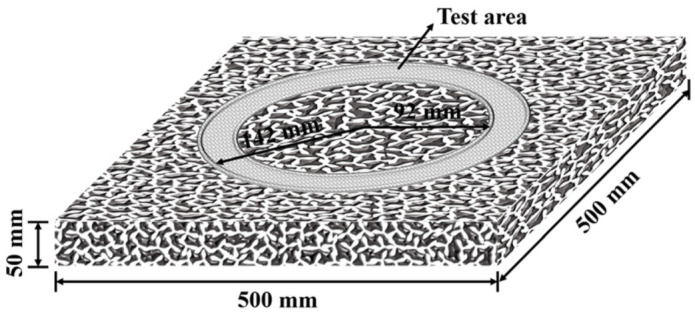
Schematic diagram of SSAM specimens.

Experiment 4: SSAM specimens were exposed to long-term wear via a self-developed three-wheel wear device (Figure 1), with every 5000 times constituting a cycle, and the wear was conducted for 10 cycles. The device was able to replicate tire wear on the road surface. Along the circular path, the average spacing between three tires was 120°, and the specimen’s surface wear was accelerated. During the test, the tire pressure on the surface of the specimen was 0.7 MPa, the tire running speed was 50 r/min, and the test temperature was 20 °C. At the end of each wear cycle, the British pendulum number (BPN) on the surface of each specimen was measured with the British pendulum. Three of each type of SSAM were made, and the BPN was taken as the average of three tests. The three-dimensional topography was measured using a pavement laser scanner (9400HD, China) with a scanning range of 30 mm × 30 mm and a scanning precision of 150 μm. Figure 2 shows the morphology of the SSAM specimen.

## 3. Results and Discussion

### 3.1. Long-Term Polishing Resistance of Aggregate

#### 3.1.1. The Attenuation Law of PSV

**Figure 3:**
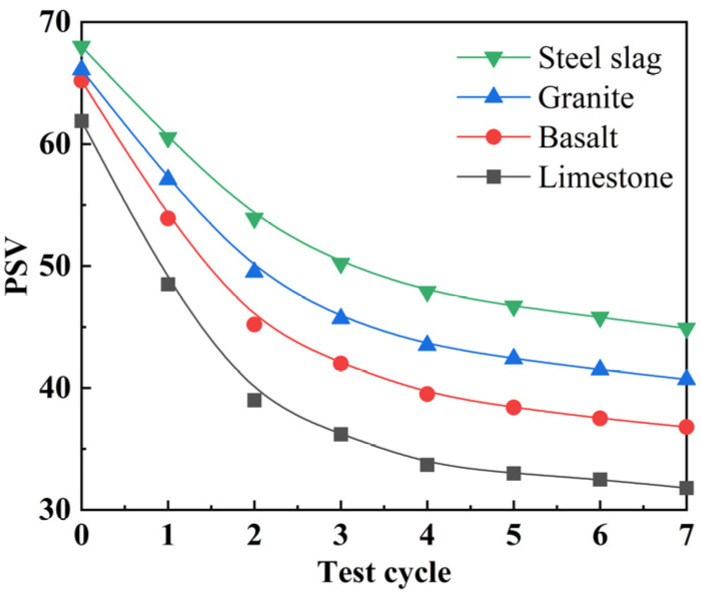
PSV attenuation curves.

Figure 3 shows the attenuation curves of aggregate PSV. It can be seen from the graph that when the polishing times were 0, the PSV of steel slag was the highest at the beginning, followed by granite, basalt, and then limestone. In the 0–2 cycles, the PSV of the aggregates declined abruptly, whereas in the 2–7 cycles, the PSV attenuation rate of the aggregates reduced progressively. The order of PSV did not change after the entire cycle of polishing, but the difference between the beginning value and the final value grew.

**Figure 4:**
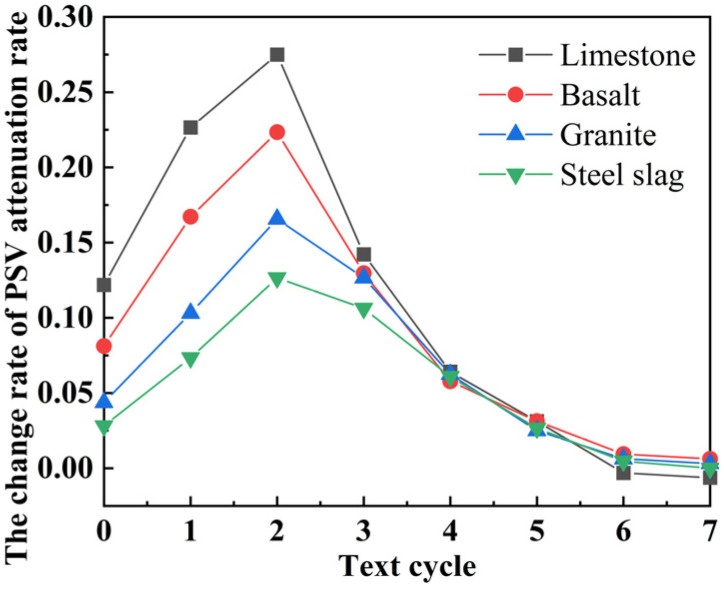
The change rate of PSV attenuation rate.

The change rate of the PSV attenuation rate with test times can be calculated using the second-order differential of PSV, as shown in Figure 4. According to the graph, PSV was in a quick attenuation stage between 0 and 2 cycles, and the change rate of PSV attenuation rate increased as polishing times increased. Limestone’s change rate of PSV attenuation rate was the greatest, followed by basalt and granite, and then steel slag. In cycles 2–7, the PSV attenuation rate continued to decelerate, and the change rate of PSV attenuation rate declined with increasing polishing times, becoming relatively constant after the fourth cycle. Compared to the natural aggregates employed in this study, steel slag had the best polishing resistance over the long term.

#### 3.1.2. Changes in Micromorphology of Aggregate Surface

**Figure 5:**
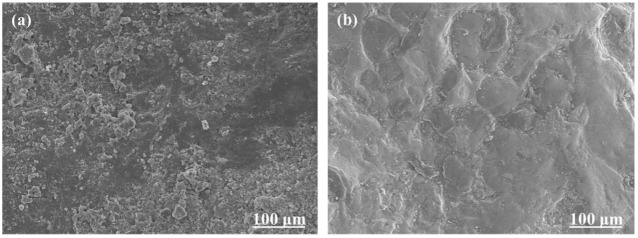
(**a**,**b**) are the SEM images of the steel slag before and after 7 polishing cycles, respectively.

**Figure 6:**
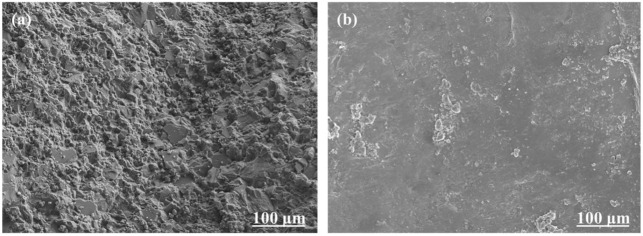
(**a**,**b**) are the SEM images of the limestone before and after 7 polishing cycles, respectively.

The steel slag with the highest polishing resistance and the limestone with the lowest polishing resistance were chosen to be photographed by SEM, capturing images of the two without and after seven polishing cycles, as shown in Figure 5 and Figure 6, respectively. The SEM pictures show that the surface morphologies of steel slag and limestone are different, but both have rough surfaces. Both of them have complex texture features and large texture depth. After seven cycles of polishing, the surface morphologies of the two objects are noticeably distinct. The surface of limestone is predominantly flat, whereas the surface of steel slag retains excellent textural depth and features.

### 3.2. Mineral Composition of Aggregates

**Figure 7:**
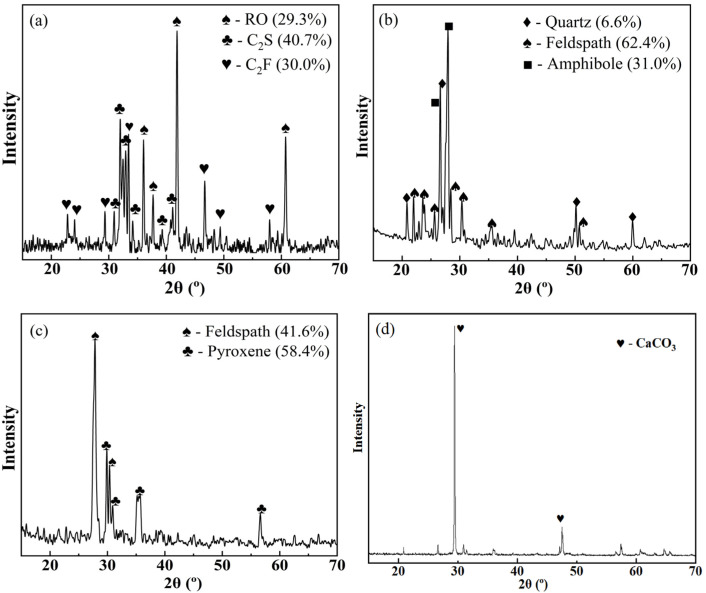
(**a**–**d**) are the XRD spectra of steel slag, granite, basalt, and limestone, respectively.

The XRD spectra of aggregates are shown in Figure 7. Figure 7a demonstrates that the steel slag was constituted of RO, C_2_S, and C_2_F in percentages of 29.3%, 40.7%, and 30.0%, respectively. RO is a continuous solid solution of divalent metal oxides and the hardest material found in steel slag. C_2_F has a lower hardness than RO, but a higher hardness than C_2_S. Figure 7b demonstrates that granite was constituted of quartz, feldspath, and amphibole in the percentages of 6.6%, 62.4%, and 31.0%, respectively. Quartz is the hardest of these minerals, whereas feldspath has the same hardness as amphibole. The analysis of Figure 7c reveals that basalt was constituted of feldspath and pyroxene in percentages of 41.6% and 58.4%, respectively. Feldspath has a higher hardness than pyroxene. The analysis of Figure 7d reveals that limestone contains only one mineral, termed calcite, which has a low hardness. Hence, limestone has poor polishing and wear resistance. The exact mineral content of the aggregate is shown in Table 2.

### 3.3. Prediction Model of Polishing Resistance of Aggregate

The Moh’s hardness of each mineral in natural aggregates was measured by Kane [28], and the nanohardness of each mineral in steel slag was measured by Wang [29]. In this paper, the nanohardness measured by Wang [29] was converted into Moh’s hardness. Kane [30] mentioned a calculation method for the average hardness of aggregates, as shown in Equation (1). According to this method, the average hardness of aggregates was calculated.

(1) $$H=\sum_{i}(d_{i}\times p_{i})$$

In Equation (1), H is the average hardness of aggregate, *d_i_* is the Moh’s hardness of each mineral that constitutes the aggregate, and *p_i_* is the mass percentage of each mineral that constitutes aggregate.

The higher hardness of the aggregate can make the aggregate maintain a good macro profile under long-term polishing to maintain a higher PSV. Therefore, steel slag and granite were more resistant to polishing than basalt and limestone. However, the PSV of aggregate is not only influenced by the aggregate macro profile but also by the aggregate surface microtexture richness.

Varied minerals in the aggregate have different hardnesses, resulting in different mineral mass losses on the aggregate surface under the same polishing impact. The minerals with lower hardness wear more, whilst the minerals with higher hardness wear less; therefore, the aggregate surface can form a secondary texture, known as differential polishing. The differential polishing principle demonstrates that an aggregate with more kinds of minerals and a greater difference in mineral hardness is more likely to have a better microtexture, resulting in a higher polishing resistance.

This paper proposed a new parameter to quantify the richness of aggregate surface microtexture called Surface Texture Richness (STR). The specific calculation method of STR is shown in Equations (2) and (3).

(2) $$m=C_{n}^{2}$$

(3) $$\overset{m}{\underset{i}{\sum }}\left[\left|d_{ia}-d_{ib}\right|\cdot \left(w_{ia}/w_{ib}\right)\right]$$

In Equation (2), *n* is the number of minerals contained in aggregate, and *m* is the combination number of two different minerals. In Equation (3), *d_ia_* and *d_ib_* are the Moh’s hardness of the two minerals, *w_ia_* and *w_ib_* are the mass ratios of the two minerals in the aggregate, and *w_ia_* ≤ *w_ib_* is required. The reason for this requirement is that if the molecule in the equation is significantly larger than the denominator, the STR will be particularly large. However, if the mineral content occupies an absolute advantage, the microtexture of the aggregate will become simple, and the STR will be small to match the actual. Considering that the bigger the difference in hardness between two minerals, the more favorable the differential polishing effect, |*d_ia_*-*d_ib_*| is employed as the weight coefficient of *w_ia_*/*w_ib_*.

Table 2 shows the mineral type and content of aggregate, the Moh’s hardness of each mineral, and the calculation results of H and STR of aggregates. The average hardness of steel slag, granite, basalt, and limestone can be observed in the table to be 6.2, 6.1, 5.7, and 3.0, respectively. The hardest aggregate was steel slag, followed by granite and basalt, whereas limestone is far softer than the other three. The STR of steel slag was significantly greater than that of granite, basalt, and limestone due to the substantial variance in hardness of the numerous minerals that compose steel slag. In addition, the STR of limestone was 0 because it was formed of a single mineral and the aggregate surface cannot undergo differential polishing. It can be concluded that the long-term skid resistance of aggregate composed of a single mineral is basically determined by the hardness of aggregates, and the effect of microtexture can be ignored.

Both aggregate hardness and aggregate microtexture have a positive correlation with aggregate PSV. Therefore, the multivariate linear regression equation between them was fitted in this research. With aggregate H and STR as independent variables and PSV as the dependent variable, a mathematical relationship was created among PSV and H and STR. The PSVP in the equation is the PSV predicted by the model, as indicated in Equation (4).

(4) $$\mathrm{PSVP}=24.830+2.250H+2.312{\mathrm{STR},\;R}^{2}=0.845$$

According to Equation (4), the coefficients of H and STR are reasonably close, but the former is substantially bigger than the latter, showing that the long-term skid resistance of aggregate depends mostly on aggregate hardness, while microtexture plays only a minor effect.

### 3.4. Long-Term Skid Resistance of SSAM

#### 3.4.1. Attenuation Law of BPN

**Figure 8:**
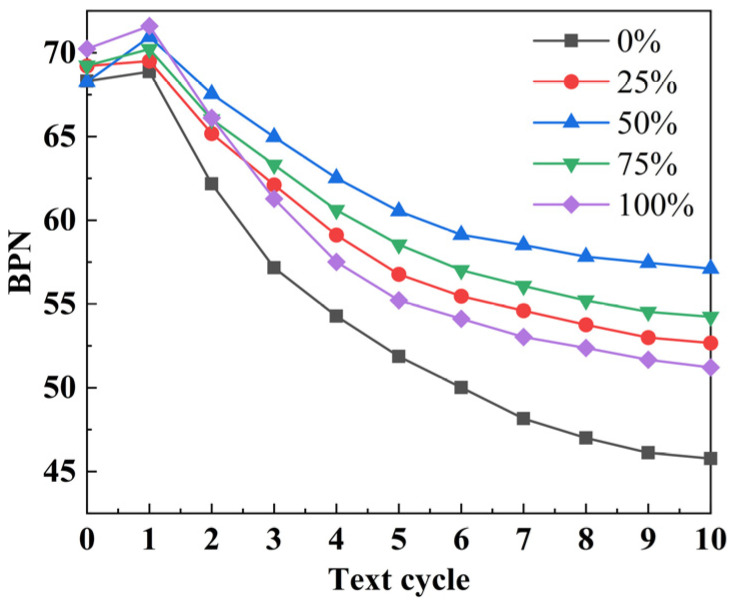
BPN attenuation curves of SSAM.

The BPN attenuation curves of SSAM are shown in Figure 8. It can be seen from Figure 8 that the addition of steel slag can increase the initial value of BPN in asphalt mixture, although there was no correlation between the amount of steel slag and the improvement in BPN initial value. The reason was that the inclusion of steel slag enhances the textural depth of SSAM, hence increasing the initial value of BPN. In the initial condition, the surface layer of aggregate is coated with a layer of asphalt film, which reduces the aggregate’s skid resistance. In addition, there was a nonlinear relationship between the increase in contents of steel slag and the enhancement of texture depth. Further inspection of Figure 8 reveals that BPN increased somewhat following the conclusion of the first wear cycle, when the asphalt film on the surface of the coarse aggregate was worn away and the coarse aggregate began to play a major role in skid resistance. The resistance to friction of aggregate is significantly greater than that of asphalt binder; hence, BPN rises [31]. After a quick fall, BPN tended to become gradually stable, and its attenuation trend was comparable to that of PSV. After the tenth wear cycle, the BPN value of SSAM with varying steel slag contents, from high to low, was as follows: 50% > 75% > 25% > 100% > 0%. There was a nonlinear relationship between steel slag content and SSAM skid resistance. The SSAM containing 50% steel slag had the best long-term skid resistance.

#### 3.4.2. Surface Texture Changes of SSAM

**Figure 9:**
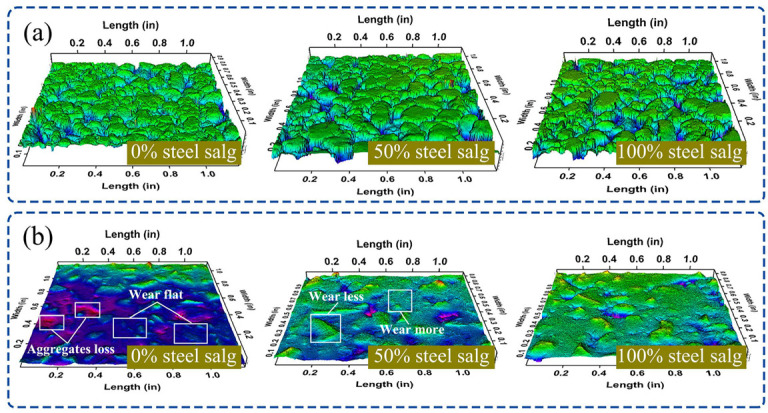
(**a**,**b**) are the surface texture of SSAM before and after 10 cycles of wear.

SSAM with steel slag contents of 0%, 50%, and 100% was chosen to study the surface texture changes before and after 10 cycles of wear, as shown in Figure 9. The initial states of the SSAM surfaces with varied slag contents were nearly identical, as shown in the figures. However, after 10 cycles of wear, the surface textures of the SSAMs with different steel slag contents were significantly different. The surface of SSAM containing 0% steel slag was significantly worn out, and the original surface structure was nearly gone. The morphology of sporadic bare aggregate particles was also round, and there was a phenomenon of aggregate shedding. SSAM with 50% steel slag content had the best surface macrotexture, and the convex and concave parts of the surface were staggered. This is because limestone has a low resistance to wear, but steel slag has a higher resistance, causing the two have distinct degrees of wear. After the combination of steel slag and limestone, a differential polishing event occurs at the macro level, which enriches the surface macrotexture of SSAM. Therefore, the SSAM containing 50% steel slag offered the greatest skid resistance. The SSAM surface with 100% steel slag content was relatively flat, and there was no visible concave section. This is because the steel slag is hard to wear away, the SSAM surface was not too badly damaged, and the wheel kept packing more of it down.

By analyzing the change of SSAM surface texture, it can be found that an increased application of high-skid-resistance aggregate in asphalt pavement is not necessarily better. The mixed use of aggregates with good and poor skid resistance can provide a differential polishing effect at the macro level, which is advantageous to enhancing the surface texture and skid resistance of asphalt pavement.

### 3.5. Long-Term Skid Resistance Prediction Model of SSAM

To further establish the mathematical relationship between aggregate’s composition and the long-term skid resistance of SSAM, an exponential model was developed to forecast the long-term skid resistance of SSAM. The macrotexture of the pavement and the polishing resistance of the aggregates influence the skid resistance of SSAM, and the macrotexture of pavement was related to the amount of steel slag and limestone. Therefore, a new dependent variable X was utilized as the independent variable in the prediction model, whereas BPN was used as the dependent variable. The calculation method of X is shown in Equation (5).

(5) $$X=\left(PSVP_{a}/PSVP_{b}\right)\cdot \left(V_{a}-V_{b}\right)$$

where PSVP_a_ and PSVP_b_ are the PSVP of steel slag and limestone, respectively, and V_a_ and V_b_ are the amounts (volume percentage) of steel slag and limestone in the coarse aggregates, respectively.

**Figure 10:**
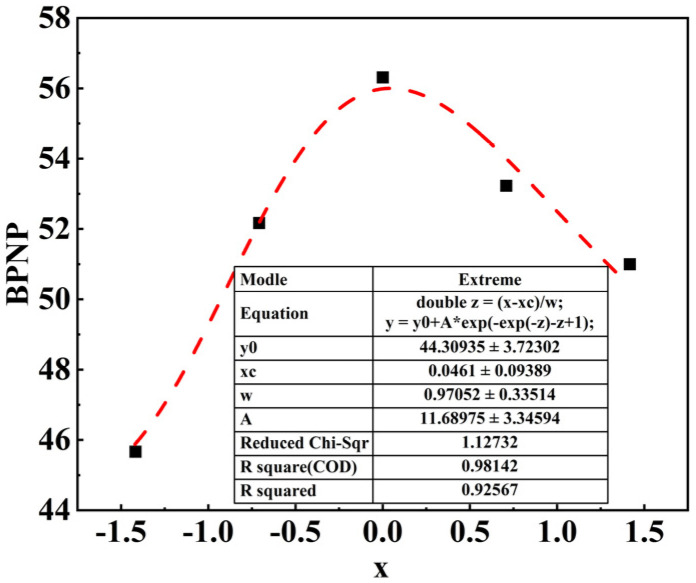
Exponential model fitting curve.

The fitting curve of the exponential model is shown in Figure 10. The specific equations of the exponential model are shown in Equations (6) and (7). BPNP in Equation (7) is the predicted BPN. The fitting results show that the correlation of the prediction model is 0.93, which has high accuracy.

(6) $$Z=\left(X-0.046\right)/0.971$$

(7) $$\mathrm{BPNP}=44.309+11.690e^{\left[\left(-e^{\left(-z\right)}-z+1\right)\right]}$$

## 4. Conclusions and Prospects

In this paper, the long-term polishing resistance of aggregates and the long-term skid resistance of SSAM were tested, and the mineral composition of aggregates was analyzed in order to establish the relationship between the mineral composition of aggregates and the long-term skid resistance of SSAM. Based on the results and discussions, the following conclusions are drawn:

(1) The polishing resistance of steel slag was superior to that of natural aggregate, with the H and STR of steel slag being superior to those of natural aggregate.

(2) The linear regression model among PSV, H, and STR can forecast the long-term polishing resistance of aggregates based on their mineral composition. The regression model indicated that the polishing resistance of aggregate correlates more strongly with aggregate hardness than with aggregate microtexture.

(3) SSAM with 50% steel slag content had superior long-term skid resistance, and there was nonlinear relationship between steel slag content and SSAM’s long-term skid resistance. The combination usage of steel slag and limestone can provide a differential polishing effect at the macro level, improve the pavement’s texture, and increase its skid resistance.

(4) The established exponential model can forecast the long-term skid resistance of SSAM based on the PSV prediction value and dose of different aggregates.

The mathematical model developed in this paper can predict the long-term skid resistance of SSAM from the mineral composition of the aggregates, but the model takes into account fewer influencing factors. In addition to hardness and microtexture, the skid resistance of aggregate is controlled by aggregate particle size, angularity, crystal structure, and crystal size. Future research will need to further investigate the relationship between aggregate PSV and these influencing factors. In addition, the research methodology in this paper can be used as a reference for long-term skid resistance studies of mixed aggregate asphalt mixtures and can also be extended for application to high-friction surface treatments (HFSTs).

## Figures and Tables

**Table 1:** Mechanical and physical properties of different aggregates.

Aggregate Type	Apparent Density (g/cm^3^)	Surface Dry Density (g/cm^3^)	Gross Volume Relative Density (g/cm^3^)	Water Absorption (%)	Crush Value (%)	Los Angeles Wear Value (%)
Steel slag	3.657	3.450	3.372	2.32	11.21	12.34
Limestone	2.717	2.686	2.667	0.69	16.67	18.11
Granite	3.035	2.952	2.885	0.79	10.87	11.95
Basalt	2.826	2.782	2.743	0.51	12.76	14.53

**Table 2:** H and STR of aggregates.

Aggregate Type	Mineral Type	Mineral Composition (%)	Mineral Hardness (d) (1–10)	Average Hardness (H)	Surface Texture Richness (STR)
Steel slag	RO	29.3	7.1	6.2	2.621
	data	40.7	5.4		
	C_2_S	30.0	6.5		
Granite	C_2_F	6.6	7.0	6.1	0.319
	Quartz	62.4	6.0		
	Feldspath	31.0	6.0		
Basalt	Amphibole	41.6	6.0	5.7	0.356
	Feldspath	58.4	5.5		
Limestone	Pyroxene	100	3.0	3.0	0

## Data Availability

The data presented in this study are available on request from the corresponding author. The data are not publicly available as the project has not been completed.

## References

[1] China Association of Metalscrap Utilization (2022). Promote Comprehensive Utilization of Steel Slag Resources, Turn Solid Waste into Resources, and Promote Green Development. http://www.camu.org.cn/feigangxiehui/article_detail.html?id=34165&code=101002&type=0.

[2] Yu D.M., Xiong R., Li S., Cong P.L., Shah A., Jiang Y. (2019). Laboratory evaluation of critical properties and attributes of calcined bauxite and steel slag aggregates for pavement friction Surfacing. J. Mater. Civ. Eng..

[3] Crisman B., Ossich G., Bevilacqua P., Roberti R. (2020). Degradation prediction model for friction of road pavements with natural aggregates and steel slags. Appl. Sci..

[4] Amelian S., Manian M., Abtahi S.M., Goli A. (2018). Moisture sensitivity and mechanical performance assessment of warm mix asphalt containing by-product steel slag. J. Clean. Prod..

[5] Liu J.Z., Yu B., Hong Q.Z. (2020). Molecular dynamics simulation of distribution and adhesion of asphalt components on steel slag. Constr. Build. Mater..

[6] Moura B.L.R., Teixeira J.E.S.L., Simão R.A., Khedmati M., Kim Y., Pires P.J.M. (2020). Adhesion between steel slag aggregates and bituminous binder based on surface characteristics and mixture moisture resistance. Constr. Build. Mater..

[7] Delgado B.G., Fonseca A.V., Fortunato E., Paixão A., Alves R. (2021). Geomechanical assessment of an inert steel slag aggregate as an alternative ballast material for heavy haul rail tracks. Constr. Build. Mater..

[8] Skaf M., Manso J., Aragón Á., Fuente-Alonso J., Ortega-López V. (2017). EAF slag in asphalt mixes: A brief review of its possible re-use. Resour. Conserv. Recycl..

[9] Logeshwari J., Sivapullaiah P.V. (2021). Physical, chemical, morphological and strength characteristics of steel slags in view of its potential application in geotechnical engineering. Mater. Today Proc..

[10] Yildirim I.Z., Prezzi M. (2015). Steel slag: Chemistry, mineralogy and morphology. Geotech. Spec. Publ..

[11] Paixão A., Fortunato E. (2021). Abrasion evolution of steel furnace slag aggregate for railway ballast: 3D morphology analysis of scanned particles by close-range photogrammetry. Constr. Build. Mater..

[12] Bessa I., Branco V.C., Soares J.B. (2014). Evaluation of polishing and degradation resistance of natural aggregates and steel slag using the aggregate image measurement system. Road Mater. Pavement Des..

[13] Ergin B., Gökalp i., Uz V.E. (2020). Effect of aggregate microtexture losses on skid resistance: Laboratory-based assessment on chip seals. J. Mater. Civ. Eng..

[14] Liapis I., Likoydis S. (2012). Use of electric arc furnace slag in thin skid-resistant surfacing. Proc. Soc. Behav. Sci..

[15] Kowalski K., Mcdaniel R.S., Olek J. (2008). Development of a laboratory procedure to evaluate the influence of aggregate type and mixture proportions on the frictional characteristics of flexible pavements. Conf. Ser. Asph. Paving Technol..

[16] Oluwasola E.A., Hainin M.R., Aziz M.M. (2015). Evaluation of rutting potential and skid resistance of hot mix asphalt incorporating electric arc furnace steel slag and copper mine tailing. Indian J. Eng. Mater..

[17] Chen J.S., Wei S.H. (2016). Engineering properties and performance of asphalt mixtures incorporating steel slag. Constr. Build. Mater..

[18] Liu C., Wang T.G. (2018). Effect of fine aggregate angularity on skid-resistance of asphalt pavement using accelerated pavement testing. Constr. Build. Mater..

[19] Skaf M., Pasquini E., Revilla-Cuesta V.V., Ortega-López V. (2019). Performance and durability of porous asphalt mixtures manufactured exclusively with electric steel slags. Materials.

[20] Pathak S., Choudhary R., Kumar A., Shukla S.K. (2020). Evaluation of benefits of open-graded friction courses with basic oxygen furnace steel-slag aggregates for hilly and high-Rainfall regions in India. J. Mater. Civ. Eng..

[21] Uz V.E., Gokalp I. (2017). The effect of aggregate type, size and polishing levels to skid resistance of chip seals. Mater. Struct..

[22] Cui P.D., Wu S.P., Xiao Y., Yang C., Wang F. (2020). Enhancement mechanism of skid resistance in preventive maintenance of asphalt pavement by steel slag based on micro-surfacing. Constr. Build. Mater..

[23] Keymanesh M.R., Ziari H., Zalnezhad H., Zalnezhad M. (2021). Mix design and performance evaluation of microsurfacing containing Electric Arc Furnace (EAF) steel slag filler. Constr. Build. Mater..

[24] Zalnezhad M., Hesami E. (2020). Effect of steel slag aggregate and bitumen emulsion types on the performance of microsurfacing mixture. J. Traffic Transp. Eng..

[25] Yildirim I.Z., Prezzi M. (2017). Experimental evaluation of EAF ladle steel slag as a geo-fill material: Mineralogical, physical & mechanical properties. Constr. Build. Mater..

[26] Wu S.P., Xue Y.J., Ye Q.S., Chen Y.C. (2007). Utilization of steel slag as aggregates for Stone Mastic Asphalt (SMA) mixtures. Build. Sci..

[27] Ferreira V.J., Vilaplana A.S., García-Armingol T., Usón A.A., Lausín-González C., López-Sabirón A.M., Ferreira G. (2016). Evaluation of the steel slag incorporation as coarse aggregate for road construction: Technical requirements and environmental impact assessment. J. Clean. Prod..

[28] Kane M., Artamendi I., Scarpas T. (2013). Long-term skid resistance of asphalt surfacings: Correlation between Wehner-Schulze friction values and the mineralogical composition of the aggregates. Wear.

[29] Wang X. (2017). Study on Composition Structure and Dissolution Performance of RO Phase from Steel Slag. Master’s Thesis.

[30] Kane M., Edmondson V. (2020). Long-term skid resistance of asphalt surfacing and aggregates’ mineralogical composition: Generalisation to pavements made of different aggregate types. Wear.

[31] Pomoni M., Plati C., Kane M., Loizos A. (2022). Polishing behaviour of asphalt surface course containing recycled materials. Int. J. Transp. Sci. Technol..

